# Prospective, observational study to assess the performance of CAA measurement as a diagnostic tool for the detection of *Schistosoma haematobium* infections in pregnant women and their child in Lambaréné, Gabon: study protocol of the freeBILy clinical trial in Gabon

**DOI:** 10.1186/s12879-020-05445-1

**Published:** 2020-09-29

**Authors:** Yabo Josiane Honkpehedji, Ayôla Akim Adegnika, Jean Claude Dejon-Agobe, Jeannot Fréjus Zinsou, Romuald Beh Mba, Jacob Gerstenberg, Raphaël Rakotozandrindrainy, Rivo Andry Rakotoarivelo, Tahinamandranto Rasamoelina, Elisa Sicuri, Norbert G. Schwarz, Paul L. A. M. Corstjens, Pytsje T. Hoekstra, Govert J. van Dam, Andrea Kreidenweiss, on behalf of the freeBILy Consortium, G. J. van Dam, P. L. A. M. Corstjens, A. S. Amoah, C. J. de Dood, M. I. Keshinro, P. T. Hoekstra, A. Kreidenweiss, N. G. Schwarz, D. Fusco, P. Klein, A. Jaeger, E. Lorenz, A. A. Adegnika, Y. J. Honkpehedji, J. C. Dejon-Agobe, R. Beh Mba, M. Mbong Ngwese, M. Nzamba Maloum, A. Nguema Moure, B. T. Meulah, R. Rakotozandrindrainy, N. Rakotozandrindrainy, M. J. Solonirina, J. Randriamaniara, R. A. Rakotoarivelo, A. Ralaizandry, M. Radomanana, M. Rakoto Andrianarivelo, T. Rasamoelina, R. Razafindrakoto, E. Siciru, C. Aerts

**Affiliations:** 1 Centre de Recherches Médicales de Lambaréné, Lambaréné, Gabon; 2 Institut für Tropenmedizin, Universität Tübingen, Tübingen, Germany; 3 Department of Parasitology, Leiden University Medical Center, Leiden, the Netherlands; 4 German Center for Infection Research (DZIF), Tübingen, Germany; 5 Department of Infectious Diseases, Division of Internal Medicine, Academic Medical Center, University of Amsterdam, Amsterdam, the Netherlands; 6 Université d’Antananarivo, Antananarivo, Madagascar; 7 Université de Fianarantsoa, Fianarantsoa, Madagascar; 8 Centre d’Infectiologie Charles Mérieux, Antananarivo, Madagascar; 9 Fundación Privada Instituto de Salud Global Barcelona, Barcelona, Spain; 10 Bernhard-Nocht-Institut für Tropenmedizin, Hamburg, Germany; 11 Department of Cell and Chemical Biology, Leiden University Medical Center, Leiden, the Netherlands

**Keywords:** Schistosomiasis, *Schistosoma haematobium*, Clinical trial, UCP-LF CAA, *Schistosoma* circulating antigen, Pregnancy, Infant, Diagnostic test, Praziquantel, Gabon

## Abstract

**Background:**

*Schistosoma* antigen detection in urine is a valuable diagnostic approach for schistosomiasis control programmes because of the higher sensitivity compared to parasitological methods and preferred sampling of urine over stool. Highly accurate diagnostics are important in low *Schistosoma* transmission areas. Pregnant women and young children could particularly benefit from antigen testing as praziquantel (PZQ) can be given to only confirmed *Schistosoma* cases. This prevents the unborn baby from unnecessary exposure to PZQ. We present here the protocol of a diagnostic study that forms part of the freeBILy project. The aim is to evaluate the accuracy of circulating anodic antigen (CAA) detection for diagnosis of *Schistosoma haematobium* infections in pregnant women and to validate CAA as an endpoint measure for anti-*Schistosoma* drug efficacy. The study will also investigate *Schistosoma* infections in infants.

**Methods:**

A set of three interlinked prospective, observational studies is conducted in Gabon. The upconverting phosphor lateral flow (UCP-LF) CAA test is the index diagnostic test that will be evaluated. The core trial, sub-study A, comprehensively evaluates the accuracy of the UCP-LF CAA urine test against a set of other *Schistosoma* diagnostics in a cross-sectional trial design. Women positive for *S. haematobium* will proceed with sub-study B and will be randomised to receive PZQ treatment immediately or after delivery followed by weekly sample collection. This approach includes comparative monitoring of CAA levels following PZQ intake and will also contribute further data for safety of PZQ administration during pregnancy. Sub-study C is a longitudinal study to determine the incidence of *S. haematobium* infection as well as the age for first infection in life-time.

**Discussion:**

The freeBILy trial in Gabon will generate a comprehensive set of data on the accuracy of the UCP-LF CAA test for the detection of *S. haematobium* infection in pregnant women and newborn babies and for the use of CAA as a marker to determine PZQ efficacy. Furthermore, incidence of *Schistosoma* infection in infants will be reported. Using the ultrasensitive diagnostics, this information will be highly relevant for *Schistosoma* prevalence monitoring by national control programs as well as for the development of medicaments and vaccines.

**Trial registration:**

The registration number of this study is NCT03779347 (clinicaltrials.gov, date of registration: 19 December 2018).

## Background

Schistosomiasis is one of most widespread human parasitic diseases worldwide [1, 2]. Infections with blood flukes of the genus *Schistosoma* are endemic in 74 countries with more than 230 million individuals being infected and 90% of infections are occurring in sub-Saharan Africa [3]. *Schistosoma haematobium* is highly prevalent in Africa [4] resulting in chronic morbidities, particularly affecting urogenital organs. Women in child-bearing age suffer two-fold from *Schistosoma* infections: directly from immune-pathological effects induced by worm infection, and indirectly from potential adverse birth outcomes during pregnancy [5].

Currently, there is no vaccine available against schistosomiasis and control is based on a single drug - praziquantel (PZQ) for chemotherapy and prevention. The 2012 WHO roadmap on neglected tropical diseases calling for schistosomiasis elimination initiated a tremendous increase in PZQ deployment [6]. This fuelled the ever-growing concern of emergence of PZQ resistant *Schistosoma*, particularly since schistosomes with reduced susceptibility have been identified sporadically in sub-Saharan Africa [7–10]. However, drug efficacy outcomes largely depend on the accuracy of the surrogate endpoint measures that reflect treatment effects in clinical trials [11]. Clinical evaluation of PZQ efficacy, of new PZQ formulations (e.g. paediatric) [12] or of new drug candidates relies on reliable diagnostic instruments to correctly judge a drug’s potency to kill *Schistosoma* infections in humans. Egg microscopy is still the reference standard widely used in schistosomiasis diagnosis but the methodology lacks sensitivity and cannot precisely measure drug efficacy due to huge variation in egg counts intrinsically associated with egg excretion that varies from day-to-day and occurs in clusters in stool [13–17]. Egg reduction rates vary considerably in a study cohort necessitating a huge sample size in clinical trials to be able to identify significant differences between the investigational product and the control. Furthermore, available PZQ efficacy data may be limited [18, 19].

Pregnant women and their infants are two vulnerable population groups, particularly in sub-Saharan Africa, who - amongst other infectious agents - are heavily exposed to *Schistosoma* infections. Infants were thought not to be exposed enough to get infected in their first months of life, however meanwhile high infection rates in infants aged as young as 3 months have been demonstrated [20]. *Schistosoma* infection causes nutritional and hematologic deficits and overt pathology caused by chronic inflammation to eggs trapped in the bladder and urogenital tissues and can lead to granulomatosis and progress to fibrosis and cancer at the late stage. Approximately 20 million women are suffering from female genital schistosomiasis (FGS) which is caused by embolized eggs in tissues of the uterus, cervix and the lower genital tract associated with bleeding and pain. FGS is a significant risk factor for HIV infection [21, 22].

Detrimental effects of maternal schistosomiasis during pregnancy on the foetus may lead to poor birth outcomes including low birth weight, preterm delivery and maternal anaemia [5, 23, 24]. Schistosomiasis is a large contributor to anaemia and undernutrition and is per se unfavourable for the developing foetus. In 2002, the WHO recommended that all pregnant women positive for *Schistosoma* should be treated with PZQ either individually or as part of mass drug administration (MDA) programs during the second and third trimester [25, 26]. Adoption of the recommendation and implementation by national disease control programs was however delayed in most African countries, due to the side-effects of PZQ and the lack of safety data in pregnant women and unborn babies. First results from randomised controlled trials with PZQ in pregnancy meanwhile have provided evidence for the safety of PZQ also in newborns [27, 28]. Nonetheless, pregnant women, unborn babies and newborns are vulnerable populations and there is a reluctancy to include them in PZQ (mass) treatment campaigns as this would imply unnecessary exposure to PZQ (even of pregnant women and infants that are not infected). In addition, logistical and financial obstacles have delayed implementation of PZQ preventive chemotherapy in pregnant women at country levels. Accordingly, innovative patient-centred approaches, combining accurate schistosomiasis diagnosis and targeted treatment during pregnancy, are needed to control schistosomiasis in this population.

### Rationale for the study

In Gabon, *S. haematobium* is the major prevalent *Schistosoma* species and *S. intercalatum* [29] is present only at a minimal level. According to the recommended WHO strategy, in 2015 a total of 343.554 individuals would have required preventive schistosomiasis treatment in Gabon alone [30]. Despite being an upper middle-income country [31], there is no effective national control program for schistosomiasis established in Gabon, but rather the National Program for neglected tropical diseases (NTD) which includes schistosomiasis and other neglected diseases. A recently published study indicated that approximately 10% of the pregnant women living in Lambaréné and Fougamou vicinities tested positive for *S. haematobium* infection with increased odds (OR1.7, 95% CI 1.03–2.82) for low birth weights of the newborns [32, 33]. As it is true for most of the observational and interventional studies on schistosomiasis, the power of the study was weakened due to the low sensitivity of the reference schistosomiasis diagnosis applied (egg microscopy), and one might assume that a considerable proportion in the control group were misclassified as negative.

Diagnostic tests that are highly sensitive and specific are essential for the detection of *Schistosoma* infections and are urgently needed in a variety of applications such as a test-and-treat strategy to control schistosomiasis in pregnancy and as tools to determine efficacy of new interventions in clinical trials. Detection of *Schistosoma* circulating antigens is becoming an important *Schistosoma* diagnostic strategy. Circulating anodic antigen (CAA) and circulating cathodic antigen (CCA) are constantly released by schistosomes into the host circulation and day to day fluctuation of CAA in serum is relatively little [34]. Furthermore, CAA levels are correlating with the number of worms [35, 36]. As CAA and CCA have been shown to clear within a few days or weeks after PZQ treatment [18, 34, 37], assays measuring antigen levels are promising techniques to more accurately assess drug efficacy. Particularly, the detection of circulating anodic antigen (CAA) by the up-converting phosphor-based lateral flow (UCP-LF) technology has proven to be a highly sensitive and specific diagnostic tool for schistosomiasis [37–39]. The high sensitivity and specificity of the UCP-LF CAA test is warranted by the uniqueness of the antigen to the *Schistosoma* genus, in combination with specific, high affinity monoclonal antibodies, a carbohydrate specific sample pre-treatment step and a unique background free reporter technology [37, 40, 41].

The overall aim of this study is to evaluate the accuracy of the UCP-LF CAA urine test for the detection of *S. haematobium* infections in pregnant women and to validate CAA as an endpoint measure for PZQ efficacy. This study is part of the *Fast and Reliable Easy-to-Use Diagnostics for Eliminating Bilharzia in Young Children and Mothers* (freeBILy) project which aims to thoroughly evaluate the use of CCA and CAA antigen tests for the diagnosis of *Schistosoma* infections in pregnant women and their newborns [42]. The freeBILy project is complemented by an additional trial performed in Madagascar that investigates a CCA-based test-and-treat strategy integrated into routine maternal and child primary health care programmes.

## Methods

### Study design

**Fig. 1:**
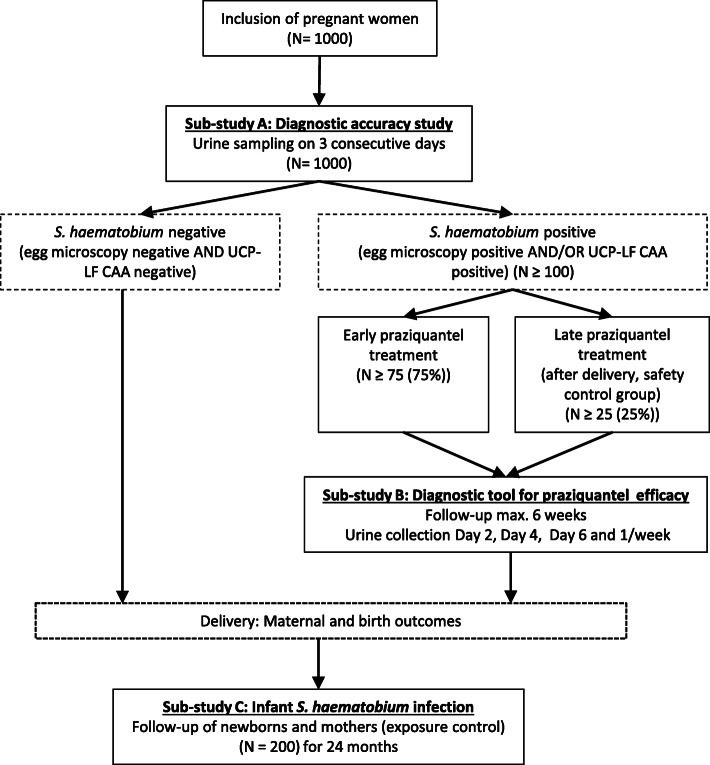
freeBILy-GABON study outline

The freeBILy Gabon-study consists of a set of 3 interlinked prospective, observational sub-studies (A-C), each targeted to assess a specific objective (see Fig. 1 for a schematic trial design, procedures and stages).

**Sub-study A** is a prospective, cross-sectional, observational study conducted in 1000 pregnant women in Lambaréné and surroundings to determine the sensitivity and specificity of the UCP-LF CAA test (index test) for the detection of *S. haematobium* infections in urine samples. Sampling will include standardized urine collection on 3 consecutive days for *S. haematobium* detection by egg microscopy and UCP-LF CAA testing; qPCR and POC-CCA will be done on one urine sample. To control for concomitant parasite infections one stool sample (within the 3 days of urine sampling) will be collected to screen for *S. intercalatum* and soil-transmitted helminths infections, as well as one blood sample will be taken for filaria and *Plasmodia* spp. detection. The blood sample will also be used to measure anti-*Schistosoma* antibodies and to provide blood cell counts and haemoglobin levels. The primary objective of sub-study A is to compare the UCP-LF CAA urine test against a composite diagnostic reference including egg microscopy, qPCR, and serology in order to determine UCP-LF CAA test performance. Sub-study A will allow to identify participants for sub-study B.

**Sub-study B** is an observational, follow-up study of at least 100 pregnant women identified in sub-study A as being infected with *S. haematobium* (by egg microscopy and/or urine UCP-LF CAA test). *Schistosoma*-infected women will be randomised 3:1 (75:25) for immediate PZQ treatment during the pregnancy (single dose of 40 mg/kg) or delayed PZQ treatment after delivery (control group). The control group will not be treated during pregnancy (to monitor “natural” CAA level fluctuation in a participant if not treated), but 6 months after delivery. The primary objective is to determine the kinetics of CAA after treatment during pregnancy compared to the control group. The control group serves also as a safety control of PZQ administration to pregnant women and exposure of their offspring (s). Therefore, both groups (treatment and control) will be actively followed-up and urine will be collected on day 2, day 4 and day 6 after PZQ treatment and then once a week until both egg microscopy as well UCP-LF CAA assay become negative but no longer than 6 weeks after PZQ treatment. The secondary objective is to collect data on safety of PZQ during pregnancy. For details on the safety investigation, see below (treatment, adverse events and safety assessment).

**Sub-study C** will follow up infants born to mothers included in sub-study A and their mothers for a maximum of 24 months (*N* = 200). This is an observational, longitudinal study with the primary objective to determine the incidence of *S. haematobium* infection and age of first *S. haematobium* infection in infants. Therefore, after delivery, mother and her infant will be asked to provide urine every 3 months until the UCP-LF CAA test becomes positive for *S. haematobium* in the infant. Children who test *S. haematobium* positive will be treated with praziquantel according to Gabonese national treatment guidelines.

In addition, a customized questionnaire addressing the knowledge, attitude and practice (KAP) of schistosomiasis of mothers and infants will be asked. Health-related quality of life (HRQoL) will also be measured by using a standardized tool: this will allow filling a wide literature gap, where only very few studies have attempted to measure HRQoL in schistosomiasis endemic areas [43]. To our knowledge, this study will be the first one collecting information on HRQoL among pregnant women in areas endemic of schistosomiasis and of other parasitic infections such as malaria.

### Study site and population

The study is planned to take place in two areas located in the center of Gabon: Lambaréné including vicinities (Bindo, Makouké and Nzilé-PK villages) and Fougamou. Both regions are endemic for *S. haematobium* with the prevalence ranging from 7 to 75%, depending on the area or the population age group [32, 44–46]. The study population are pregnant women and their newborns.

### Ethical considerations

The study protocol and informed consent forms (ICF) were approved by the National Ethics Committee of Gabon (PROT N°039/2018/SG/CNE) and by the Institutional Ethics Committee at CERMEL/Gabon (N° 017/2018). Written informed consent will be sought from any volunteer or the legal guardian of minor volunteers before any study procedure will be performed.

### Recruitment of participants, data and sample collection

Prior to enrolment, the study will be explained to the pregnant women – and legal guardian if minor - presenting to the antenatal care centres (ANC) in Lambaréné (Albert Schweitzer and Regional Georges Rawiri Hospitals). Those willing to participate will be provided with the informed consent form (ICF) for signature for themselves and for their unborn child. Particular emphasis is given to information on *Schistosoma* transmission, diagnosis, associated pathologies and risk factors for *Schistosoma* infection in particular, and to intestinal helminths in general. Inclusion criteria are pregnancy at gestational age between 16 and 30 weeks (based on last date of menses), providing signed ICF, willing to deliver in one of the two maternities in the study areas and no plans to move out of the study area during the following 24 months. Exclusion criteria are reporting of complicated previous pregnancy, known chronic infections or diseases as HIV, HBV, HBC infections. Following the ICF process, a KAP questionnaire will be administered to collect standard demographic, health history, obstetrical data and information on schistosomiasis related knowledge, attitude and practice.. Economic and health-related quality of life (HRQoL) questionnaire will be included as well. The questionnaire will be used to measure HRQoL among all pregnant women at enrollment and to monitor HRQoL among the positive women over time. For this, the HRQoL questionnaire will be administered again to positive women 1 week after enrollment when test results are available and treatment provided (at A4) and a few months later, well after treatment completion (at C2). Physical examination will be done by the study physician; participants will be asked to provide urine (midstream urine will be collected in a clean container), stool and blood samples for parasite diagnostics and health care service. At delivery, newborns will be weighted, and gestation age will be recorded. The newborn’s anthropometry parameters, Apgar score at 1 and 5 min, maternal bleeding volume, placenta quality and weight will be collected. For details on study visits over time with respect to the study phases and details of sample collection see Additional file 1.

### Diagnostics

#### Investigational product: UCP-LF CAA test

The index test is the UCP-LF strip test detecting and quantifying CAA levels. LF strips can be analysed with POC care readers [47] or, as used in this study, with a multistrip benchtop reader (Upcon; Labrox Oy, Turku, Finland) capable of analysing 20 strips at a time. The UCP-LF CAA test was developed at Leiden University Medical Center (Leiden, The Netherlands) [38, 48]. So far, the tests are produced in-house at LUMC in a batch-wise manner (ca. 2000 tests per batch), fully quality-controlled using standardized reference samples, ensuring specificity and sensitivity. A dry reagent format is available that can be stored and transported at ambient temperature [39]. Here, 417 μl of fresh or stored (− 20 °C) urine will be tested by the UCP-LF CAA urine test following published procedures [48] including a concentration step (centrifugal Amicon Ultra 0.5 mL filters with a 10 kD cut-off; Merck Millipore) after pre-treatment of the urine with 1/6 volume of 12% trichloroacetic acid and (UCAA*hT*417 format) [49]. All measurements will include a set of CAA-spiked standards into a negative urine to determine the threshold of positivity that is set to ≥2 pg/ml.

### Comparator diagnostics

#### Urine filtration and egg microscopy

*Schistosoma* egg detection by microscopy is commonly used for the diagnosis of schistosomiasis as recommended by WHO and diagnostic procedures will follow standardized protocols [50]. Urine (10 ml) will be passed through a 12 μm filter (Whatman Nucleopore) using a syringe. The filter will then be placed on a glass slide and read under a light microscopy (10x magnification). Every slide will be read by two independent microscopists. The test will be considered positive if at least one *S. haematobium* egg is found and egg counts will be given (N eggs/10 ml urine).

#### qPCR

*Schistosoma* egg DNA will be detected by real-time PCR (qPCR) on urine samples following a published protocol [51]. We will use the established *Schistosoma* genus-specific primers (Ssp48F and Ssp124R) and the dual labelled probe Ssp78T to amplify a 77-bp fragment of the internal transcribed spacer-2 (ITS2) sub-unit. An internal control will be added to monitor efficient DNA extraction. DNA extraction from urine and qPCR assay will be performed.

#### Serology

Antibodies against *S. haematobium* will be measured using a lateral flow assay (UCP-LF antibody assay) that is based on the same technology as the UCP-LF CAA test and can be evaluated using the same UCP Labrox reader. The serology test is designed to detect antibodies against soluble egg antigen (SEA) and soluble crude cercarial antigen preparation (SCAP) in sera of participants [37, 52].

### Additional diagnostics

#### POC-CCA

The point-of-care (POC) CCA urine test is a commercially available POC rapid test for the detection of CCA in urine (Rapid Medical Diagnostics, South Africa). This LF immuno-chromatography test delivers qualitative detection of active *Schistosoma* infection, and is recommended and generally applied to detect *S. mansoni* infections performing less in *S. haematobium* settings [53, 54]. It is ideally suited for field conditions as it does not require laboratory facilities or highly trained personnel.

### Microscopy for concomitant parasite infections

To control for the impact of concomitant other parasite infections on the accuracy of UCP-LF CAA, stool samples will be investigated for *S. intercalatum* (as well as any other *Schistosoma* species) and for other highly prevalent helminths such as *Ascaris lumbricoides*, *Trichuris trichuria*, Hookworms, and *Strongyloides stercoralis*. Stools will be examined by Kato-Katz and coproculture techniques [53, 55]. Therefore, approximately 50 mg of fresh stool will be processed. Microscopy reading (10x magnification) is done by two independent readers and numbers of eggs per gram of stool will be reported according to WHO guidelines [53, 56]. Remaining stool will be stored for later confirmation by respective qPCR [51]. Blood samples will be examined for infections with malaria parasites (*Plasmodium falciparum*, non-falciparum) and filarial parasites (*Loa loa*, *Mansonella perstans*) by standard diagnostic procedures (Giemsa-stained thick blood smears, saponin leucoconcentration assay followed by microscopy, respectively) [57, 58].

### Treatment, adverse events and safety assessment

Women infected with *Schistosoma* (positive by egg microscopy and/or UCP-LF CAA) will be treated with PZQ either during pregnancy (as recommended by WHO) or after delivery. Single dose PZQ (40 mg/kg) will be administered by an unblinded member of the study team under observation. Safety of PZQ administration in women will be assessed verbally within 24 h (after 1 h and 24 h later) to record all adverse events occurring after drug administration. All adverse events will be assessed by the study physician, following local guidelines and will be graded as mild, moderate or severe. All investigators (study physicians and lab technicians) will be blinded to the treatment allocation. Other parasitic infections will be treated following national treatment guidelines. This work will be done in collaboration with the Gabonese National Program for the Fight against NTDs who will contribute to the PZQ supply.

### Additional outcomes

Maternal health outcomes, maternal anaemia (defined as Hb < 11 g/dl) at inclusion and at delivery will be investigated. In offspring, birthweight (with low birthweight defined as weight at birth < 2.5 kg) and small for gestational age (used as an indicator for possible intrauterine growth restriction) will be investigated. In addition, the exposure to PZQ during pregnancy will be evaluated in infants at the age of 12 and 24 months. Time to sit, crawl, stand and walk, incidence of illnesses and vaccine coverage during the follow-up will be recorded.

### Data safety and monitoring board

An independent data safety and monitoring board (DSMB) has been established which will review safety data in the sub-study B during the study.

### Sample size calculation

Analysis of the UCP-LF CAA (UCAA*hT*417) test performance will be compared against a composite diagnostic reference (i.e. by latent class analysis). Assuming a 10% egg positivity rate in the study population [32], about 1000 pregnant women should be tested to obtain 100 *Schistosoma* positive women. Assuming further a sensitivity of the index test (UCP-LF CAA) between 80 and 100% with 100 infected women, statistical precision (two-sided 95% confidence limit) of ±7.8% will be achieved if a sensitivity of 80% is observed and a lower limit of 96.4% if a sensitivity of 100% is observed. Due to the limited sensitivity of the egg detection test, to estimate the specificity, only study participants negative for reference tests should be included. Furthermore, assuming a true *Schistosoma* prevalence of 20% (based on a conservative estimate of 50% sensitivity of the egg test) and assuming a false positive rate of 20% of the other two reference tests (PCR and UCP-LF antibody test), at least 500 women will enter the naïve estimate of specificity. This will lead to a statistical precision of between ±3.5 and ± 4.3% depending on the observed specificity. For sub-study B, the analysis is based on the assumption of a non-inferiority delta of 0.10, identical probabilities of positivity and negativity at the end of the sub-study B for both tests (UCP-LF CAA and egg microscopy) and a discordance of 12%, with 100 pregnant women, the power will be 80% to show non-inferiority (type 1 error: 2.5% one-sided).

### Data management and statistical analysis

All data will be collected on paper forms which will be entered into a REDCap database [59]. Initial data will be double entered in REDCap to ensure their accuracy. Each participant included into the study will receive a unique identifier. The case report from (CRF) is the source documents for personal data, clinical data and laboratory result sheets will be considered as source documents for laboratory results reported in the CRF.

Analysis of the UCP-LF CAA (UCAA*hT*417) test performance will be compared against a composite diagnostic reference by using Latent Class Analysis or other Bayesian approaches which will allow to compare the estimated sensitivity, specificity, positive predictive value (PPV), negative predictive value (NPV) and the accuracy values for detecting *Schistosoma* in the sample. The model will be fitted to determine the Bayesian Information Criteria (BIC), Akaike information Criteria (AIC), the Chi square test and the standardized Residuals.

To assess the effect of PZQ treatment on birth weight, Analysis of covariance (ANCOVA) will be used. This is a fundamental statistical method of analysis for both randomised and non-randomised studies. It is a regression analysis which includes parameter estimates for observed covariates in addition to the treatment effects we are ultimately interested in estimating.

## Discussion

There is no vaccine against schistosomiasis so far, and MDA of PZQ remains important for the control of schistosomiasis but is not sufficient when moving towards the goal of schistosomiasis elimination. The ideal test for test and treat strategies would be an easy to use point of care test, which is highly sensitive and specific for all *Schistosoma* species (without the necessity to distinguish between the species as the treatment is the same for all species) and used material that does not require invasive procedures (e.g. urine or saliva instead of blood). Microscopic methods lack sensitivity and require a lot of resources time and trained personnel. Serology cannot distinguish between an acute, ongoing and past infection and PCR methods require advanced microbiological facilities and capabilities. Antigen tests, however can provide easy to use, field suitable tests [19, 60, 61]. There is a need of sensitive and accurate *Schistosoma* diagnostics to support communities in need and also control programs. The freeBILy Gabon study will comprehensively evaluate the performance of the UCP-LF CAA laboratory test with the focus on pregnant women and its extended usefulness as a read-out measure for PZQ efficacy.

The UCP-LF-CAA test is highly sensitive and specific and also performs well for urogenital schistosomiasis. Unfortunately, this test is not currently available as a point of care test. The assessment of this test in pregnant women in a *S. haematobium* endemic regions with weekly follow up of initially positive pregnant women as well as in infants to validate the CAA antigen test as an endpoint for measuring PZQ efficacy. Determining the true efficacy of PZQ against different *Schistosoma* infections is important for schistosomiasis control programs. It informs on the susceptibility of local *Schistosoma* infections towards PZQ as well as providing information for future efficacy trials for paediatric formulations of PZQ. Furthermore, using the UCP-LF-CAA test minimizes sample size in future efficacy trials as CAA levels are relatively constant in serum or urine, whereas the number of eggs is subjected to highly random daily fluctuations. The UCP-LF-CAA test clearly demonstrates that prevalence of active *Schistosoma* infections is underestimated by a factor of up to ten when compared to egg microscopy. Especially in low-endemic settings, the UCP-LF-CAA test is superior over parasitological methods. This test can easily identify individuals with very low levels of infection as well as early infections and is well-suited for determining efficacy (or even failure) of treatment. The test is ready to be taken up by industry for upscaling and deployment at large scale. So far, there is no alternative for evaluating long-term MDA programmes, and moving towards post-MDA surveillance or elimination.

Beyond the study, this work will prepare for the development of CAA rapid diagnostic tests, that can play a role in the future as a test-based schistosomiasis treatment strategy in pregnancy in endemic regions. Furthermore, the study tackles a neglected disease (schistosomiasis) and provides a concept to overcome the neglect of pregnant women and small children by schistosomiasis control programmes.

## Trial status

Recruitment of study participants has started in April 2019 and is expected to be finished by April 2021: Follow-up of sub-study B is expected to be finished by July 2021 and of sub-Study C by the end of 2022.

## Supplementary information

**Additional file 1.** Overview of study visits over time with respect to the study phase and details of sample collection, describes the study phases and procedures.

## Data Availability

Not applicable.

## Abbreviations

ANC: Antenatal care
CAA: Circulating anodic antigen
CCA: Circulating cathodic antigen
CERMEL: Centre de Recherches Médicales de Lambaréné
CRF: Case report form
FGS: Female genital schistosomiasis
GA: Gestational age
Hb: Hemoglobin
HIV: Human Immunodeficiency Virus
LUMC: Leiden University Medical Center
MDA: Mass drug administration
NTD: Neglected Tropical Disease
POC-CCA: Point-Of-Care Circulating Cathodic Antigen
PZQ: Praziquantel
qPCR: (Semi-)quantitative real-time polymerase chain reaction
UCP-LF: Up-converting phosphor technology based lateral flow
WHO: World Health Organisation
