# Supplementary material for: Prospective, observational study to assess the performance of CAA measurement as a diagnostic tool for the detection of Schistosoma haematobium infections in pregnant women and their child in Lambaréné, Gabon: study protocol of the freeBILy clinical trial in Gabon
Source: BMC Infect Dis. 2020 Sep 29;20:718. doi: 10.1186/s12879-020-05445-1 (PMC7523491; doi:10.1186/s12879-020-05445-1)
Supplement: Supplementary file 1 — Additional file 1. Overview of study visits over time with respect to the study phase and details of sample collection, describes the study phases and procedures. [file 12879_2020_5445_MOESM1_ESM.pdf]

Additional file 1: Overview of study visits over time with respect to the study phase and details of sample collection

Timeline:		Baseline			W1	W2	W3	W4	W5	W6	W7		D	D+7	Q2	Q3	Q4	Q5	Q6	Q7	Q8	CO
		PREGNANCY (pregnant woman followed up)											POST-DELIVERY (mother and child followed up)									
Visit name:		**Sub-study A (sensitivity and specificity of the UCP-LF-CAA test)**																				
		**A1**	**A2**	**A3**	**A4**								**A5**	**A6**								
					**Sub-study B (CAA levels)**																	
					**B4-B7**	**B8**	**B9**	**B10**	**B11**	**B12**	**B13**											
													**Sub-study C (early *S. haematobium* infections in infants)**									
													**C1**		**C2**	**C3**	**C4**	**C5**	**C6**	**C7**	**C8**	**C9**
**Mother**	Urine (ml)	25	25	25	25	25	25	25	25	25	25		15		15	15	15	15	15	15	15	
	Blood (ml)	10																				
	-Serum (ml)	5																				
	-EDTA (ml)	5																				
	Stool	X								X												
	PZQ (40 mg/kg)				X										X							X
**C**	Urine (ml)														5	5	5	5	5	5	5	5

Ch: Child, Tt: Treatment, W: Week, D: Delivery, D+7: Delivery plus 7 days, Q: Quarter (Q1 starts with delivery), CO: Close-out
